# Spatially Resolved Thermometry of Resistive Memory Devices

**DOI:** 10.1038/s41598-017-14498-3

**Published:** 2017-11-10

**Authors:** Eilam Yalon, Sanchit Deshmukh, Miguel Muñoz Rojo, Feifei Lian, Christopher M. Neumann, Feng Xiong, Eric Pop

**Affiliations:** 1 Department of Electrical Engineering, Stanford University, Stanford, CA 94305 USA; 2 Department of Materials Science & Engineering, Stanford University, Stanford, CA 94305 USA; 3 Precourt Institute for Energy, Stanford University, Stanford, CA 94305 USA; 4 Present Address: Department of Electrical & Computer Engineering, University of Pittsburgh, Pittsburgh, PA 15261 USA

## Abstract

The operation of resistive and phase-change memory (RRAM and PCM) is controlled by highly localized self-heating effects, yet detailed studies of their temperature are rare due to challenges of nanoscale thermometry. Here we show that the combination of Raman thermometry and scanning thermal microscopy (SThM) can enable such measurements with high spatial resolution. We report temperature-dependent Raman spectra of HfO_2_, TiO_2_ and Ge_2_Sb_2_Te_5_ (GST) films, and demonstrate direct measurements of temperature profiles in lateral PCM devices. Our measurements reveal that electrical and thermal interfaces dominate the operation of such devices, uncovering a thermal boundary resistance of 28 ± 8 m^2^K/GW at GST-SiO_2_ interfaces and an effective thermopower 350 ± 50 µV/K at GST-Pt interfaces. We also discuss possible pathways to apply Raman thermometry and SThM techniques to nanoscale and vertical resistive memory devices.

## Introduction

Information storage and memory devices based on the change of resistance (i.e. resistive memories or memristors) hold several key advantages over contemporary charge-based memory devices. Such memory devices are two-terminal resistors that retain their resistance state as a function of the applied voltage or current. Several technologies can be included under the general term of “resistive memory”^1,2^, such as phase change memory (PCM)^3,4^, resistive random access memory (RRAM)^5,6^, and conductive bridge RAM (CB-RAM)^7^. Memristive devices are important not only for memory and storage applications; they are also being extensively studied as computing elements for neuromorphic architectures^2,8^.

The resistive switching in RRAM devices is based on the formation and rupture of conductive filaments in thin metal oxides, like HfO_2_. In PCM, a nanoscale volume of chalcogenide material (like Ge_2_Sb_2_Te_5_) can be SET to a crystalline (low resistive) state and RESET to an amorphous (high resistive) state using electrical pulses. Self-heating and the local temperature play a major role in the principle of operation of both PCM^4^ and RRAM^9,10^. Many of the advantages (e.g. energy efficiency improved with scaling)^11,12^ and shortcomings (e.g. reliability)^13^ of these technologies stem from their inherent dependence on self-heating. Therefore, understanding the energy and heat dissipation mechanism is vital for the evaluation, design and optimization of all such future technologies. However, experimental techniques to measure nanoscale device temperature are challenging and scarce^10,14,15^. In particular, spatially resolved measurements revealing energy dissipation mechanism are required for better understanding of the device physics^16,17^.

Here we combine Raman thermometry and scanning thermal microscopy (SThM) to measure the spatially resolved temperature rise in resistive memory devices. This powerful combination of temperature mapping techniques has been previously used on GaN nanowires^18^, but it is applied here to resistive memory devices for the first time. We present temperature-dependent Raman spectroscopy of thin films for two of the most commonly used RRAM oxides: HfO_2_ and TiO_2_, and for the PCM material Ge_2_Sb_2_Te_5_ (GST). We then show an experimental measurement of the temperature profile in a Joule-heated PCM device, providing important insights into its operation. Finally, we discuss how Raman and SThM can be used to measure the local temperature rise in vertical and other nanoscale RRAM and PCM device geometries.

## Raman Spectroscopy of RRAM Oxide Films

Raman spectroscopy measures the shift in inelastically scattered light, directly corresponding to phonon energy (*ℏ*ω) and temperature (*T*). Stokes (anti-Stokes) lines are due to photons scattered at lower (higher) energy than the incident laser, due to phonon emission and absorption, respectively. (More Raman spectroscopy details are given in Supplementary Information Section 1.) Fundamentally, most RRAM oxides have poor Raman signal due to weak absorption (ultra-thin films with large band gap) and low degree of crystallinity. However polycrystalline oxide film regions or the programmed RRAM filament could be expected to have different Raman signals, and are yet to be studied. For example, Raman spectroscopy has been previously used to study stoichiometry, defects, and particularly oxygen vacancies in crystalline^19^ and nano-crystalline^20^ oxides. The Raman spectra of oxide powders, single crystals, and thick films have previously been reported^21–23^, but here we present the first temperature-dependent Raman spectra of nanoscale thin films which are relevant for RRAM devices.

**Figure 1:**
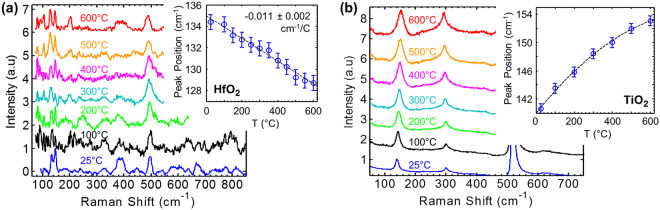
Temperature dependent Raman spectra of RRAM oxide nanoscale thin films. Raman spectra of crystallized 50 nm thin films of (**a**) HfO_2_ and (**b**) TiO_2_, measured at temperatures ranging from 25 °C to 600 °C. Spectra are vertically offset for clarity. Insets show peak position shift with temperature of a selected mode, error bars represent 95% confidence bounds of Lorentzian peak fitting. The HfO_2_ was sputtered onto Pt (50 nm) on sapphire substrate and then annealed at 600 °C for 2 hours. All measured peaks above 110 cm^−1^ are assigned to monoclinic phase of HfO_2_ (as confirmed by XRD)^24^. The TiO_2_ was sputtered onto SiO_2_ (90 nm) on Si substrate and annealed at 400 °C for 1 hour. The peak at ~141 cm^−1^ (25 °C) is assigned to anatase TiO_2_ and the other peaks are from the Si substrate.

Figure 1 shows the temperature-dependent Raman spectra of two of the most common RRAM oxides: (a) HfO_2_ and (b) TiO_2_. The 50 nm thick films were sputtered onto Pt/sapphire and SiO_2_/Si substrates, respectively, and did not show any measurable Raman features in their (as-deposited) amorphous state. After annealing (see Methods) the films crystallized, exhibiting Raman signals of the monoclinic (HfO_2_) and anatase (TiO_2_) phases. The insets show the temperature dependence of a selected mode. The B_g1_ monoclinic HfO_2_ mode^24^ (~134 cm^−1^ at 25 °C) shows typical frequency downshift with temperature at rate of ~ −0.011 cm^−1^/C. The E_g_ anatase TiO_2_ mode (~141 cm^−1^ at 25 °C) however shows anomalous frequency increase with temperature. This trend was previously reported and explained via strong contribution of the quartic anaharmonicity^23^. For the practical purpose of device thermometry it is sufficient to have a well-defined temperature response of the Raman mode, either positive or negative.

We note that temperature-dependent Raman data are presented in this section for nanoscale oxide films for the first time. However, obtaining sufficient signal in nanoscale RRAM *devices* is challenging, but could be addressed using signal enhancement techniques such as surface- or tip- enhanced Raman spectroscopy (SERS^25^ or TERS^26,27^). In addition, an optically transparent electrode such as graphene^28^, indium tin oxide (ITO), or a very thin (e.g. sub-10 nm) metal can be used to measure vertical device structures. In the next section we focus on Raman thermometry of PCM, which exhibits a sufficiently strong signal to demonstrate (spatial) device thermometry.

## RAMAN Spectroscopy of PCM

### Raman Spectroscopy of GST Films

Raman spectroscopy has previously been used to characterize nanoscale PCM films^29–31^. Unlike oxides, GST absorbs photons in the range of visible excitation lasers (~500–650 nm) due to its smaller band gap (~0.6 eV). In addition, much of the GST volume in a typical PCM cell is crystalline, thus Raman thermometry can be readily applied to PCM devices. Moreover, Raman spectroscopy can be used to identify phase and presence of defects in GST as outlined below.

**Figure 2:**
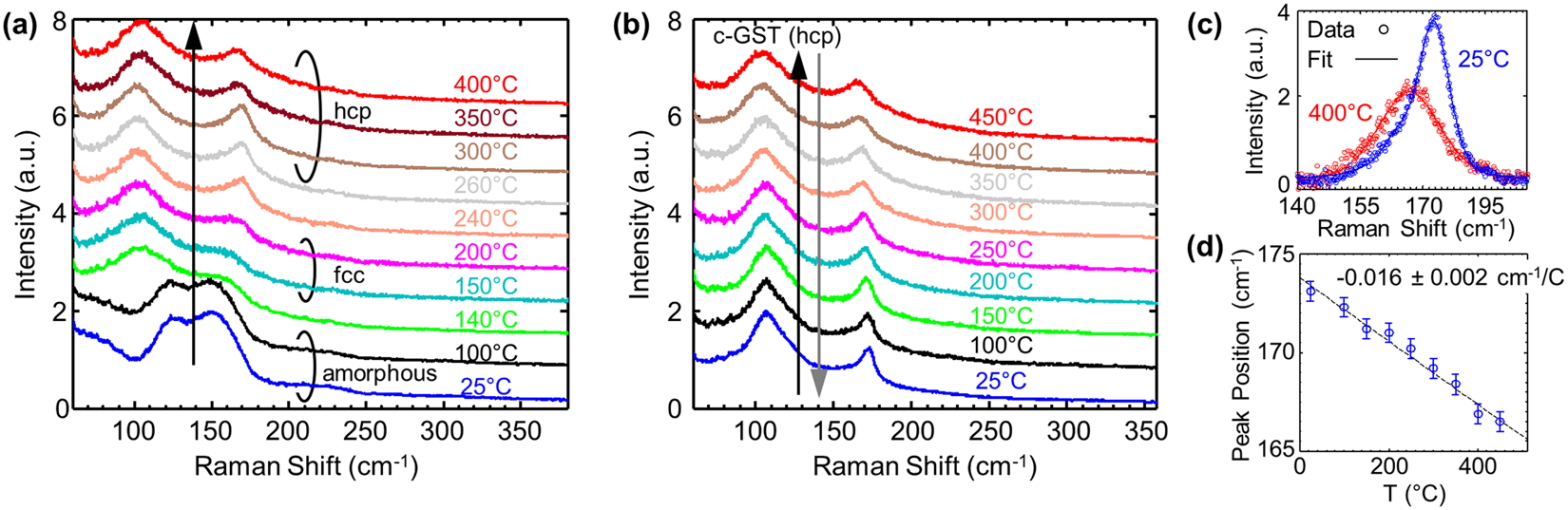
Temperature dependent Raman spectra of 20 nm thin GST film. (See Methods). (**a**) Raman spectra during the first heating cycle on a hot stage from 25 °C to 400 °C. As-deposited, the GST is amorphous, but after heating the film starts to crystallize first to the fcc phase and then to the hcp phase. (**b**) Temperature dependent Raman spectra of the stable hcp phase GST at temperatures ranging from 25 °C to 450 °C. Similar spectra were obtained for cooling (not shown). (**c**) Example of measured (symbols) and fitted (lines) peak for a selected mode at 25 °C and 400 °C in the hcp phase. (**d**) Peak position shift vs. temperature of the selected mode shown in (**c**). Error bars represent 95% confidence bounds of Lorentzian peak fitting.

Figure 2 shows the temperature dependent Raman signal of 20 nm thin sputtered GST films (see Methods). Some previous studies have performed such measurements on 50 to 150 nm thick GST films^32–34^ but we are unaware of temperature-dependent Raman data for GST films of the thickness studied here (~20 nm), which are more relevant for modern memory devices. Figure 2(a) shows the first heating cycle on a hot stage from 25 °C to 400 °C. The as-deposited film is amorphous (a-GST) and starts to crystallize (c-GST) at ~140 °C, first to the face centered cubic (fcc) phase and then at ~240 °C to the hexagonal closest pack (hcp) phase. For assignment of the various Raman peaks to GST modes please see ref.^29^. Upon cooling to room temperature and heating back up to 450 °C the film remains in its stable hcp phase, as shown in Fig. 2(b). Figure 2(c) shows the measured (symbols) and fitted peak of a selected mode at stage temperatures of 25 °C (blue, ~173 cm^−1^) and 400 °C (red, ~168 cm^−1^) and Fig. 2(d) shows the peak position downshifting vs. stage temperature from 25 °C to 450 °C.

### Raman Spectroscopy of GST Devices

**Figure 3:**
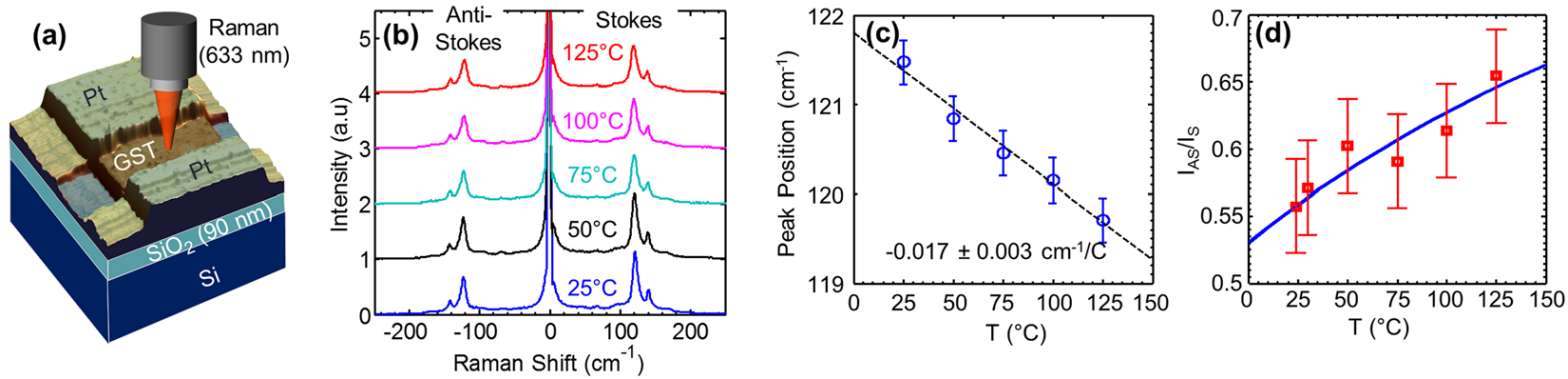
Temperature dependent Raman spectra of lateral GST device. (**a**) Device and measurement setup. The GST channel (*W* = 10 µm, *L* = 5 µm) is patterned on top of Pt electrodes and capped with PMMA (see Methods). (**b**) Stokes and anti-Stokes Raman spectra of patterned GST device on a hot stage at temperatures from 25 °C to 125 °C. These are dominated by Te modes^35^ with much higher intensity than GST modes. (**c**) Peak shift with temperature of a selected mode (at ~120 cm^−1^). (**d**) Anti-Stokes to Stokes intensity ratio vs. temperature of the selected mode shown in (c).

Thanks to its material and phase selectivity, Raman spectroscopy can be used to map GST films and PCM devices for phase analysis. However, here we uncover that patterned and processed GST *devices* exhibit Raman spectra that are different from blanket deposited GST *films*. Figure 3(a) schematically shows the Raman measurement of a lateral GST device with Pt contacts, and device fabrication details are provided in the Methods section.

Figure 3(b) shows the Stokes and anti-Stokes Raman signal of the GST device on a hot stage from 25 °C to 125 °C. The stage temperature was kept below the highest temperature during processing (PMMA bake at 180 °C) in order to avoid changes to the GST channel and/or contacts. It is evident that the Raman signal of the GST device is dominated by two intense peaks at ~120 and ~140 cm^−1^, which are not present in the GST film spectra (Fig. 2). These peaks were present in the GST device immediately after lift-off, before spin-coating the PMMA capping. The intensity of these peaks is significantly larger than the GST film peaks shown in Fig. 2.

These intense peaks (~120 and ~140 cm^−1^) are known from previous studies as Te peaks^35^. We have also measured similar peaks in uncapped GST films after exposure to high laser power and formation of dark spots in the film (Supplementary Information Section 2). These peaks were also measured in other sets of lateral GST devices which were capped with different oxides^36^. Such oxide-capped devices switched for many cycles (~10^5^) with good on/off ratio (~10^2^) and low switching energy (<20 pJ). These Te peaks are likely associated with surface oxidation of the GST during processing, resulting in formation of GeO_x_ and SbO_x_, and precipitation of Te. The former is also evident from X-Ray photoelectron spectroscopy (XPS) of the dark spots at the GST surface (Supplementary Information Section 2), while the latter is evidenced in our measured Raman spectra.

The position of the lower (~120 cm^−1^) Te peak has strong temperature dependence, as shown in Fig. 3(c) which allows us to use it efficiently as a thermometer. Raman spectroscopy can then be used to extract temperature in two different ways; one via the calibration of peak shift vs. temperature on the hot stage^37,38^ and the other directly from the anti-Stokes to Stokes (AS/S) intensity ratio^39^:

1 $$T=\frac{\hslash {\omega }_{{\rm{ph}}}}{{k}_{{\rm{B}}}}{[3\mathrm{ln}(\frac{{\omega }_{{\rm{L}}}+{\omega }_{{\rm{ph}}}}{{\omega }_{{\rm{L}}}-{\omega }_{{\rm{ph}}}})-\mathrm{ln}({I}_{{\rm{AS}}}/{I}_{{\rm{S}}})]}^{-1}$$

where *T* is the phonon temperature (in K), *ω*_ph_ and *ω*_L_ are the phonon shift and laser frequency, respectively, *I*_AS_/*I*_S_ the anti-Stokes to Stokes intensity ratio, and *k*_B_ is the Boltzmann constant. The advantage of the AS/S method is that in principle it does not require a calibration procedure and that the temperature can be obtained with a single measurement. This is attractive for spatial temperature mapping of devices since the temperature map can be obtained in a single map scan, whereas for the peak shift method a calibration measurement is needed and at least two map scans must be obtained^38^ (e.g. one with and the other without bias). Nonetheless, the uncertainty in the measured temperature of our GST devices using the AS/S method was large compared with the peak shift method, as described below.

The symbols in Fig. 3(d) show the measured AS/S intensity ratio of the spectra from Fig. 3(b) after baseline subtraction and Lorentzian peak fitting. The error bars are obtained from the standard deviation of ~30 measurements (carried out at ambient *T* = 25 °C). The blue line represents eq. (1). The main drawback of the AS/S method is the uncertainty in the temperature evaluation, because the extraction of intensity from peak fitting is less accurate than the extraction of peak position. (Similar errors are encountered when using the *area* under the peak instead of the peak intensity.) Moreover, the *absolute* temperature *T* (≥300 K) is obtained from the intensity ratio with relative error ~15% in our case. Since we are interested in measuring the *change* in temperature (Δ*T* ~ 100 K above room temperature) the relative error in Δ*T* is three times larger, or nearly ~ 50%. At the same time, the relative error in Δ*T* for the peak shift method is less than ~15% [e.g. see Fig. 3(c)]. Therefore, in the remainder of this study we use the peak shift method to map the temperature of the device, following the procedure outlined in ref.^38^ for aligning the two maps. We note that temperature sensitivity of the AS/S method should improve for phonon modes at higher frequencies (Supplementary Information Section 3).

## PCM Device Thermometry

### Measurement Technique

**Figure 4:**
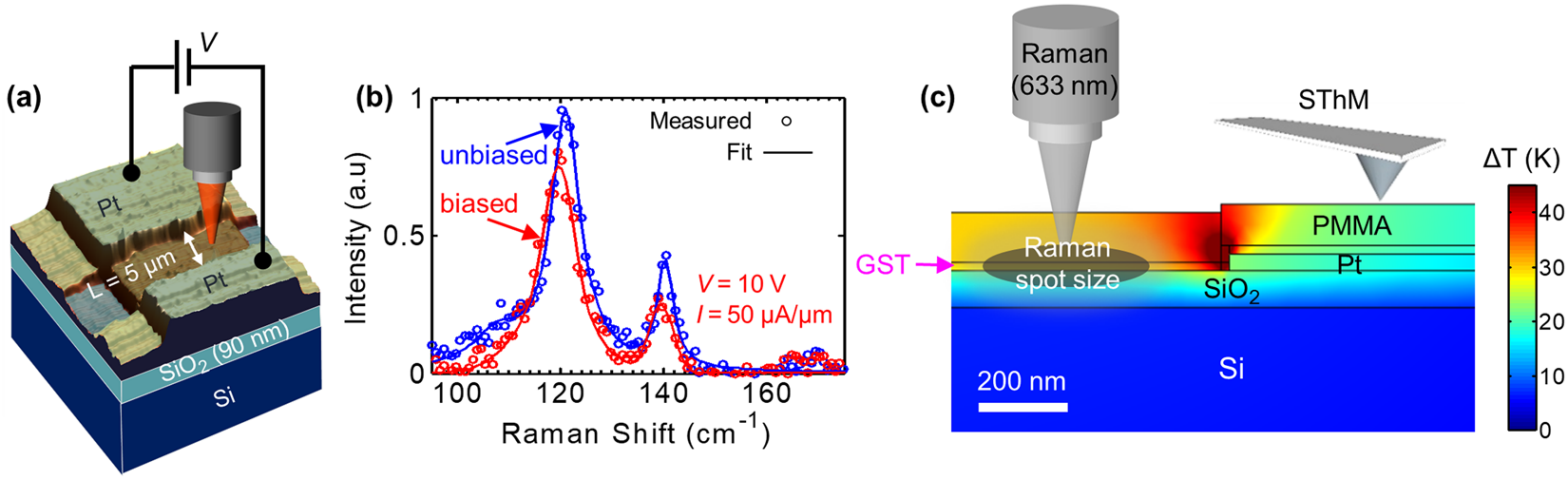
Thermometry of a lateral PCM device. (**a**) Measurement setup: Raman (and SThM) acquired during device operation with self-heating. (**b**) Measured (symbols) and fitted (lines) Raman spectra of the GST at the center of the channel with electrical bias (red: *V* = 10 V, *I* = 0.5 mA) and without bias (blue). (**c**) Simulated cross-section temperature profile of the device near the contact, highlighting the temperature measured by Raman (directly on GST film with Gaussian laser spot size) and SThM (top surface of PMMA capping layer).

Figure 4(a) displays the schematic of the Raman thermometry measurement applied to a lateral GST device. We spatially mapped the device with 0.25 µm step size, measuring the Raman spectra at each point with and without electrical bias. The temperature is extracted by converting the peak shift (with electrical bias and self-heating) to temperature via the calibration shown in Fig. 3(c). Figure 4(b) shows the measured (symbols) and fitted (lines) Raman spectra at the center of the GST channel with (red) and without (blue) electrical bias. The Te peaks at ~120 and 140 cm^−1^ are clearly visible, as well as their shift with self-heating. Raman features at ~105 and 170 cm^−1^ may correspond to GST hcp phase, but their signal is significantly smaller.

We also carried out scanning thermal microscopy (SThM) measurements on the same devices. SThM is an atomic force microscopy (AFM) based technique that uses a thermo-resistive probe to acquire nanoscale topographic and thermal images simultaneously^40,41^. Unlike Raman, SThM measures only the surface temperature and requires additional calibration (see Supplementary Information Section 4), however it provides nearly AFM-like spatial resolution (<100 nm) and is therefore used here to provide complementary insight into PCM device thermometry.

Figure 4(c) shows the simulated cross-sectional temperature profile in our lateral GST device and contact. Three-dimensional (3D) finite element simulations were carried out using COMSOL Multiphysics®. To better understand the measurements, we illustrate the Raman and SThM instruments with the cross-sectional simulated temperature. The Raman method measures the temperature of the GST channel but its signal is averaged across the Gaussian laser beam spot (here ~ 400 nm). Inherently the SThM has better spatial resolution with a thermal exchange radius of <100 nm^42^, yet it measures the temperature at the top surface of the PMMA capping layer which spreads the heat. Simulations therefore predict slightly different temperature profiles for the Raman and SThM measurements. We note that the SThM is operated here in DC mode and is not calibrated to output temperature directly, rather it outputs a relative signal. We then use Raman thermometry (with same power input conditions) to calibrate the SThM to the temperature in the middle of the PCM device, where the GST temperature (measured by Raman) and the top PMMA temperature (measured by SThM) are similar [see cross-sectional temperature rise in Fig. 4(c)]. This calibration is made possible by the fact that the device temperature is uniform across several microns in the center of the GST channel, a region larger than the laser spot size.

The main advantage of the Raman measurement is its material selectivity which allows a differential measurement of materials in the laser path, thus enabling even atomic scale resolution in the cross-plane direction^43^. However, not all materials have a usable Raman signal and the spatial resolution is diffraction-limited (unless a signal enhancement technique such as TERS^26^ is utilized). SThM on the other hand has nearly AFM-like spatial resolution, but measures the temperature at the top surface rather than the direct temperature of the material of interest. The temperature sensitivity of the SThM can be better than that of the Raman method, but in order to convert the SThM signal (voltage) to temperature, the Raman method was used for calibration, as outlined above. Both techniques are limited in measuring *vertical* RRAM devices; the top electrode might block the Raman signal and laterally spread the temperature profile measured by SThM. Nonetheless, the combination of both techniques could become a very powerful tool to study the power dissipation in cases where temperature plays a major role in device operation^2,3,5,10^. A practical solution is to measure lateral devices or vertical devices having transparently thin top electrode.

### Power Dissipation in Lateral PCM Device

**Figure 5:**
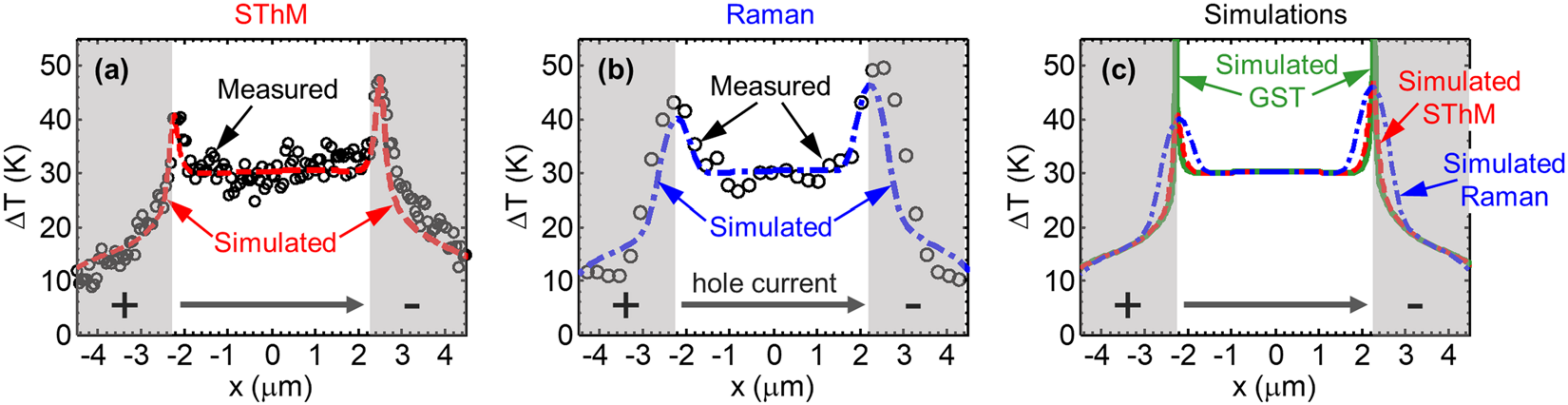
Measured vs. simulated temperature rise. Temperature rise along GST channel in fcc phase (black symbols) measured by (**a**) SThM and (**b**) Raman thermometry. The gray zones mark the contact regions. The simulated temperature rise fitted by finite element modeling for SThM (red dashed line) and Raman (blue dash-dot line) are also shown in (**a**) and (**b**) respectively. (**c**) Simulated temperature rise of the GST channel (green solid line), the SThM (top of PMMA surface averaged across thermal exchange radius^42^) same as in (**a**) and Raman (GST with Gaussian laser spot size) same as in (**b**). Black arrows mark hole current flow direction.

Figure 5 compares the temperature profile along the device channel (including the contacts) measured by (a) SThM and (b) Raman to the simulated temperature rise with very good agreement. The full device temperature maps are shown in Supplementary Information Sections 5 and 6. Figure 5(c) shows the simulated temperature rise of the GST channel (green) as well as the profile that would be measured by SThM (red) and Raman (blue). Figure 5 highlights how the power dissipation in our devices is revealed via both Raman thermometry and SThM, with varying degrees of spatial accuracy. By inspecting the measured temperature profiles in Fig. 5 and the fitting parameters used in the simulation (summarized in Table I of Supplementary Information Section 9), several conclusion can be drawn as follows.

First, it is evident that significant heat is generated at the contacts^44^. Temperature artifacts due to the presence of step heights at the edge of the GST-Pt contacts are negligible here compared with the observed interfacial heating as discussed in Supplementary Information Section 6. It appears that the large contact resistivity of the GST film on the Pt contact (GST is deposited on top of the Pt electrodes) leads to highly localized power density, mainly at the edge of the electrode (also evident in the SThM maps shown in Supplementary Information Section 5). We also carried out transfer length method (TLM) measurements to directly extract the contact and sheet resistance (Supplementary Information Section 7) which served as inputs to the simulations. However, the temperature peak at the Pt-GST edge suggests that much of the contact voltage may be dropped at the imperfect GST step coverage of the Pt electrodes. To account for this, we set the GST resistivity at the Pt sidewall to be 20х larger than the bulk GST resistivity, leading to correct fitting in our simulations. The temperature rise of the 5 µm long GST channel suggests that much of the power dissipates there, yet the temperature peaks at the contacts due to the high power *density* there.

Second, given the power input and the measured temperature rise we can extract the thermal boundary resistance (TBR) of the GST-SiO_2_ and GST-Pt interfaces to be 28 ± 8 and 25 ± 9 m^2^K/GW respectively. Since the Si and SiO_2_ thermal properties are well known, and given the insensitivity to the bulk GST thermal conductivity (see Supplementary Section 9) the GST-SiO_2_ TBR acts as a single fitting parameter to the temperature rise at the center of the PCM device. The obtained value is in agreement with the TBR of the GST-SiO_2_ interface previously measured^45^ by the time domain thermo-reflectance (TDTR) technique, but it is extracted here within a functioning PCM device for the first time. The measured TBR of the GST-SiO_2_ interface is equivalent to a Kapitza length of ~50 nm of SiO_2_, which accounts for more than 25% of the total thermal resistance in our device. Importantly, the relative contribution of the TBR is expected to increase and dominate as devices are scaled down in size^46^.

Third, we also observe a clear asymmetry in heating at the two contacts, with the higher temperature at the edge of the grounded electrode. This contact heating asymmetry is due to a combination of thermoelectric and thermionic effects^17,47^ as confirmed by reversing the current flow direction. We extract an effective thermopower *S* = 350 ± 50 µV/K, which includes both thermoelectric and thermionic effects at the GST-Pt contact. Similar values were reported for GST in the mixed amorphous and fcc phase with Pt^48^ and with TiW^49^ contacts slightly above room temperature, after annealing up to ~ 150 °C. Following anneal at higher temperature (>200 °C) and higher degree of crystallization, the thermopower is expected to drop significantly (below 50 µV/K)^48,49^. The higher temperature at the grounded contact is also consistent with the location of damage after device breakdown, shown in Supplementary Information Section 8. The asymmetric heating highlights the importance of designing the voltage polarity of PCM programming pulses, to take advantage of thermionic and thermoelectric effects^50^. We note that our test devices are lateral and larger than state-of-the-art PCM devices^51–54^; however the physical insights are valid and highly valuable: the power generation is dominated by electrical contacts and the heat dissipation is limited by thermal interfaces in nanoscale devices^44^.

## Conclusion

In summary, in this study we have laid the fundamental basis for thermometry of RRAM and PCM data storage devices. We presented the first measurements of thermal Raman signatures in nanoscale films of HfO_2_ and TiO_2_, and we used spatial mapping of temperature (with both Raman and SThM) to provide physical insight into the operation of PCM devices. Our approach takes advantage of the benefits of each technique, e.g. selectivity (Raman) and high spatial resolution (SThM), and can be extended to a wide variety of devices. We uncover significant heating at the contacts, suggesting that power dissipation is often dominated by electrical contact resistance. Contact heating is asymmetric, depending on current flow direction, showing that thermoelectric effects must be taken into account when designing PCM programming pulses. We also extract the TBR of the GST-SiO_2_ interface and find that it is equivalent to ~ 50 nm thick SiO_2_, contributing much of the device thermal resistance. The role of both electrical contacts and thermal interfaces will only become more dominant as devices are scaled to sub-50 nm dimensions. Uncovering the spatial distribution of temperature rise in such self-heated memristive devices is essential for their understanding, and their future design and integration.

## Methods

Raman measurements were carried out on a Horiba Labram Evolution HR using a 633 nm laser with an 1800 l/mm grating. The red laser is chosen here since it provides Raman signal comparable to other laser lines in our system (e.g. 532 nm) and allows the measurement of anti-Stokes signal by using a volume Bragg grating optical filter at 633 nm. All measurements were done in air.

HfO_2_ films were deposited by reactive sputtering from an Hf target in an Ar:O_2_ (7:3) plasma at 4 mTorr, with a forward RF power of 150 W, at room temperature onto thin Pt films (50 nm) on a sapphire substrate. The Pt/sapphire substrate was used to enhance the Raman signal of the HfO_2_ film. TiO_2_ films were sputtered from a Ti target in an Ar:O_2_ (14:1) plasma at 5 mTorr onto SiO_2_ (90 nm) on Si substrates. To obtain Raman signal the films were crystallized by annealing: the TiO_2_ for 1 hour at 400 °C and the HfO_2_ for 2 hours at 600 °C, both in air.

Blanket GST films (20 nm) discussed in the section “Raman Spectroscopy of GST Films” were sputtered onto SiO_2_ (90 nm) on Si substrates, immediately followed (without breaking chamber vacuum) by sputtering 20 nm SiO_2_ to prevent oxidation when later exposed to air. GST devices (section “Raman Spectroscopy of GST Devices”) were prepared as follows. First, contacts and pads were defined by photolithography. Contact separation defined channel lengths *L* = 2 to 20 µm. The 40 nm Pt contacts (with 2 nm Ti adhesion layer) were then deposited by e-beam evaporation followed by patterning of channels (of widths *W* = 1 to 10 µm) with e-beam lithography, sputtering of 20 nm GST and lift-off. Devices were capped by spin coating ~150 nm poly(methyl methacrylate) (PMMA) to prevent oxidation, then baked on a hot plate at 180 °C for 10 minutes (in air) to crystallize the GST film.

The SThM measurements were carried out in passive mode and under DC bias. Measured PCM devices were Joule heated electrically by applying constant voltage to the contact pads for ~10 minutes during the SThM scan. The SThM thermal probe model used in this work is a DM-GLA-5 from Anasys®, which is made of a thin Pd layer on SiN.

## Electronic supplementary material

Supplementary Information
