# Supplementary material for: Spatially Resolved Thermometry of Resistive Memory Devices
Source: Sci Rep. 2017 Nov 10;7:15360. doi: 10.1038/s41598-017-14498-3 (PMC5681698; doi:10.1038/s41598-017-14498-3)
Supplement: Supplementary file 1 — Supplementary Information [file 41598_2017_14498_MOESM1_ESM.pdf]

Spatially Resolved Thermometry of Resistive Memory Devices

Eilam Yalon[1], Sanchit Deshmukh[1,†], Miguel Muñoz Rojo[1,†], Feifei Lian[1], Christopher M. Neumann[1], Feng Xiong[1,2], and Eric Pop[1,3,4,*]

[1]*Department of Electrical Engineering, Stanford University, Stanford, CA 94305, USA.* [2]*Present address: Department of Electrical & Computer Engineering, University of Pittsburgh, Pittsburgh, PA 15261, USA.* [3]*Department of Materials Science & Engineering, Stanford University, Stanford, CA 94305, USA.* [4]*Precourt Institute for Energy, Stanford University, Stanford, CA 94305, USA.* [*]*E-mail: epop@stanford.edu*

[†]*Authors contributed equally*

Supplementary Information

Table of Contents

1. Raman spectroscopy
2. Raman signal of patterned GST devices
3. Frequency dependence of the anti-Stokes to Stokes intensity ratio
4. Scanning thermal microscopy (SThM)
5. SThM images
6. Temperature maps
7. Transfer length method (TLM)
8. PCM device breakdown
9. Parameters used in COMSOL simulation

1. Raman spectroscopy

Raman spectroscopy measures the shift in inelastically scattered light, directly corresponding to phonon energy ($\hbar\omega$) and temperature (T). Stokes (anti-Stokes) lines are due to photons scattered at lower (higher) energy than the incident laser, due to phonon emission and absorption, respectively. Thus, the Raman scattering process directly probes the phonon modes in the sample and provides information on the atomic structure, while also being sensitive to strain[1], doping[2], defects[3], and temperature[4,5]. The sensitivity to temperature arises from phonon mode softening (peak position downshift) as well as increased phonon scattering (peak broadening) with increasing temperature. In addition, the anti-Stokes to Stokes intensity ratio depends on the phonon population which is directly related to temperature[6]. The capability of measuring nanoscale features (such as individual carbon nanotubes)[7] combined with the sensitivity to temperature result in a potentially attractive technique for RRAM and PCM thermometry. Other details of the Raman measurements carried out in this work are presented in the Methods section of the paper.

2. Raman signal of patterned GST devices

We studied the origin of the Raman features found in our patterned GST devices which show a different spectra compared with blanket deposited GST (see Figs. 2 and 3 in the main text). The Raman spectra of our patterned GST devices is shown in Fig. S1(a) (red) and includes two prominent features: one at ~122 cm^{-1} and the other at ~142 cm^{-1} which are clearly associated with the presence of Te [Fig. S1(e-g)][8]. We compare the patterned GST Raman spectra with that of other GST films that most likely oxidized during laser-induced heating [Fig. S1(b)] and during electrical heating [Fig. S1(d)] as well as uncapped Sb_2Te_3 (partially oxidized at the surface, Fig. S1(c)). These GST and Sb_2Te_3 films were also measured by XPS and show evidence of surface oxidation (Fig. S2).

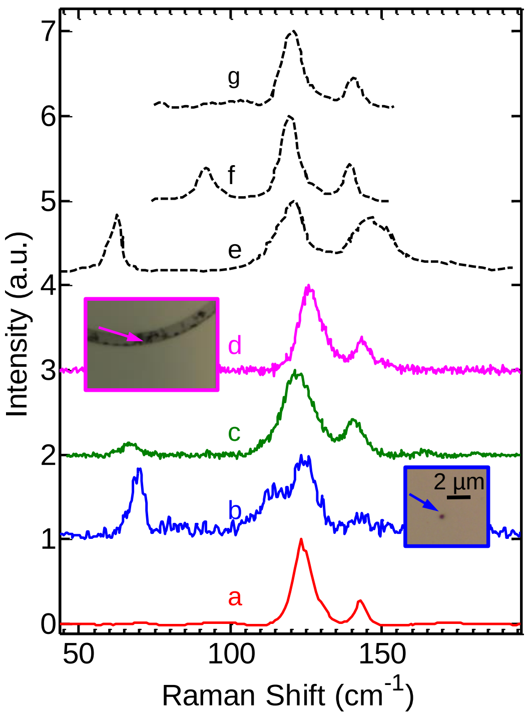

Figure S1 | Raman signatures of oxidized GST, Sb_2Te_3 and Te. Raman spectra of: (a) Patterned and finalized GST device described in the main text, after lift-off, (b) dark spot formed in uncapped GST film (inset, blue) following Raman measurement at high laser power (~10 mW). (c) Sb_2Te_3 uncapped film, (d) dark spot formed in uncapped GST circular device (inset, purple) after showing resistance changes induced by electrical pulses, (e) amorphous Te[8], (f) trigonal z-axis of Te[8], and (g) trigonal x-axis of Te[8].

Figure S2 shows X-Ray photoelectron spectroscopy (XPS) data of uncapped GST device (20 nm) thick) before and after a short Ar sputtering etch. The pre-sputter results represent the GST surface. Both Ge and Sb show native oxide peaks, whereas Te does not. Post-sputtering (i.e. a few nm into the film) the Sb_2O_3 + Sb_2O_5 peak decays and Ge + GeO_x feature narrows, suggesting the native oxide is only present at the top few nm of the film. We note that the sputtering can also induce some damage to the film, so these results are mostly qualitative. Overall, Fig. S1 and Fig. S2 suggest that surface oxidation of GST results in formation of native Ge and Sb oxides as well as Te precipitates. These Te precipitates dominate the Raman signal.

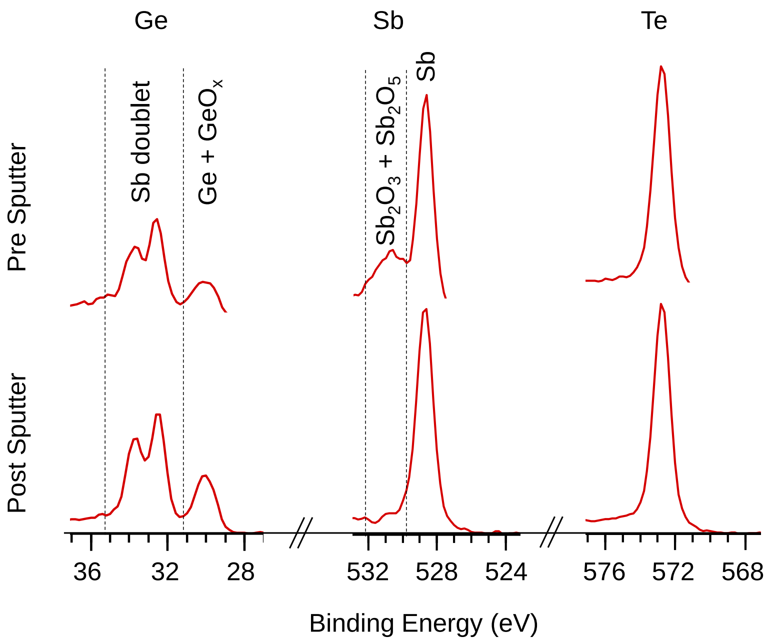

Figure S2 | XPS of uncapped (oxidized) GST. Pre-sputter (top panel) represents the GST surface, showing features of native Ge and Sb oxides as well as (un-oxidized) Te. The post-sputtering measurement (bottom panel) probes a few nm into the film, but might induce damage at the surface. The Sb_2O_5 + Sb_2O_3 feature decays and Ge + GeO_x feature narrows post-sputtering, suggesting Sb and Ge are oxidized only at the very top surface.

3. Frequency dependence of the anti-Stokes to Stokes intensity ratio

The temperature in our experiment is measured by comparing the Raman *peak shifts* in an electrically biased sample to a calibration measurement on a hot stage. The anti-Stokes to Stokes (AS/S) Raman intensity ratio can also be used to probe temperature, without the need for a calibration measurement (on a hot stage) and/or a reference measurement (without electrical bias). In our experiment the uncertainty in temperature measured by I_{AS}/I_S is significantly larger than the one measured by the peak shift method. One reason is that the uncertainty in measured (and fitted) intensity is larger compared with the measurement and fitting of (spectral) peak position. In addition, we illustrate in Fig. S3 that the temperature sensitivity in the AS/S method is poor for low wavenumbers (in our experiment the peaks are located at ~120 and 140 cm^{-1}). We note that for materials with Raman modes at higher frequency, the AS/S method could potentially be used with lower uncertainty. On the other hand, the AS signal intensity degrades with increasing mode frequency due to the decrease in phonon absorption probability.

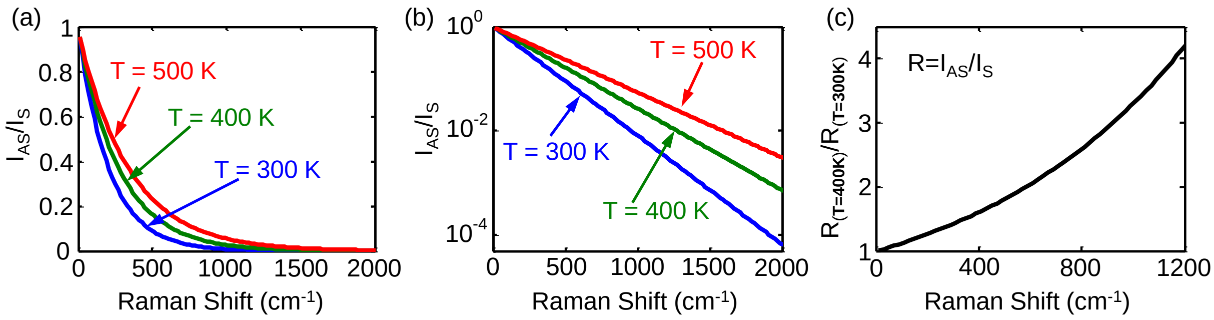

Figure S3 | Anti-Stokes to Stokes (I_{AS}/I_S) signal sensitivity. Calculated I_{AS}/I_S vs. Raman shift (peak position) at T = 300 K (blue), 400 K (green), and 500 K (red) in (a) linear and (b) log y-axis. The temperature sensitivity improves with increasing wavenumber, but the I_{AS} signal intensity decreases. (c) Sensitivity to temperature rise of ~ 100 K above room temperature; plot shows the ratio between R = I_{AS}/I_S at 400 K and 300 K vs. Raman shift of the measured mode.

4. Scanning thermal microscopy (SThM)

Scanning thermal microscopy (SThM) is an atomic force microscopy (AFM) based technique that uses a thermo-resistive probe to acquire nanoscale topographic and thermal images simultaneously[9,10]. When the probe is brought into contact with the sample surface, heat is exchanged between the surface and the probe. These temperature changes are correlated with the electrical resistance of the probe, which is measured using a Wheatstone bridge. The SThM has two main operating modes: one active, in which the probe is heated up and acts as both the heater and the thermometer, and the other passive, in which the probe is only used as the temperature sensor. The probe or sample heating can be carried out by DC or AC currents, which allow multiple ways of measuring and processing data. The SThM technique has been used to measure the thermal properties of films[11,12], nanowires[13,14], and devices[15]. In this study, the PCM device is Joule-heated in DC mode and the probe is used passively for sensing the local temperature rise. The SThM signal calibration (mV to K) is done via the Raman temperature measurement and we assume this calibration is the same on both areas of the GST: on top of the contact and on top of the channel (all capped by ~ 150 nm PMMA).

5. SThM images

Figure S4 displays SThM maps at varying input power and the corresponding AFM topography map. The heating maps show significant heating at the contact edges.

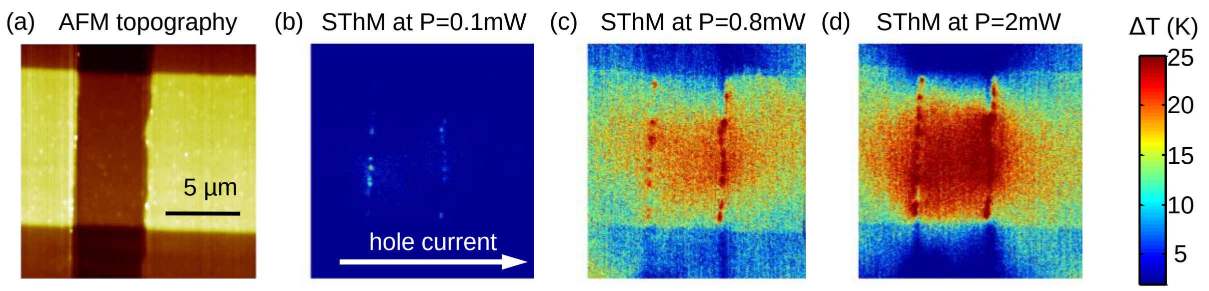

Figure S4 | AFM and SThM. (a) AFM topography map of the GST device described in the main text. Scanning thermal microscopy (SThM) maps of the same GST device with varying power inputs: (b) P = 0.1 mW, (c) P = 0.8 mW, and (d) P = 2 mW. The SThM heating map is calibrated via the Raman temperature measurement to obtain temperature values. Arrow in (b) indicates hole current flow direction for all 3 maps shown. The thermal maps show significant heating at the contact edges.

6. Temperature maps

We obtained temperature maps of the GST devices by Raman and SThM. Representative temperature profiles along the device (obtained by averaging temperature across device width) are shown in Fig. 4 of the manuscript. Figure S5 below compares measured and simulated temperature maps obtained by (b,c) SThM and (e,f) Raman. The (a) AFM topography map and (d) Raman intensity map are shown as reference to locate the channel and contact areas. Figure S6 displays a reference SThM scan when the device is unbiased (no Joule heating) showing only small artificial temperature rise (< 1 K) as a result of topography changes across the device. The step-height artifact is reduced here thanks to the surface planarization achieved by the PMMA capping layer.

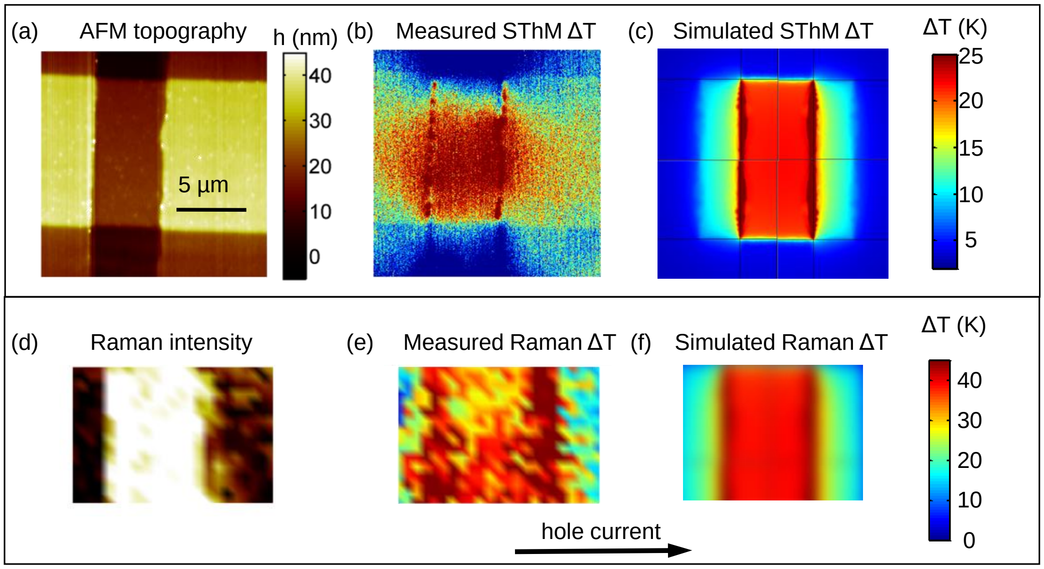

Figure S5 | Temperature maps: SThM, Raman and simulations. (a) AFM topography map of the GST device described in the main text. (b) Measured and (c) simulated SThM map of the same device at input power P = 2 mW. (d) Intensity map of the Raman mode at ~122 cm^{-1}. (e) Measured and (f) simulated Raman temperature map of the same device at input power P = 3.6 mW. Arrow indicates hole current flow.

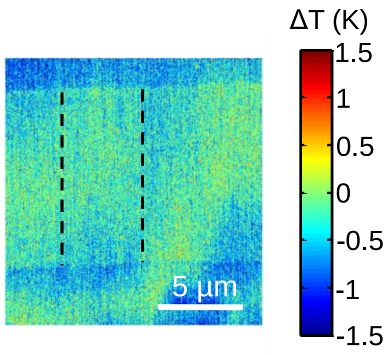

Figure S6 | Reference SThM map. Measured SThM map of the device when it is unbiased (no Joule heating) under the same probe sensing conditions of the maps shown in Fig. S5. The map shows small changes in the temperature < ~ 1 K as a result of topography changes or other artifacts. Black dashed lines indicate the edges of the electrodes.

7. Transfer length method (TLM)

Figure S7 displays TLM measurements of our GST films, showing the electrical resistance vs. channel length to obtain contact and sheet resistances. It is evident that in our lateral GST devices, for channels shorter than ~ 5 µm the contacts account for more than 50% of the total resistance, and therefore most of the power will be dissipated at the contacts. The extracted resistivity of the GST $\rho \approx 35$ mΩ·cm agrees with previous studies[16] given the annealing temperature (180 °C for 10 minutes, leading to fcc phase) used here. The *contact resistivity* could not be reliably evaluated from the measured *contact resistance* due to the poor coverage of the GST film on the Pt contact sidewall and the resulting current crowding at the contact edge.

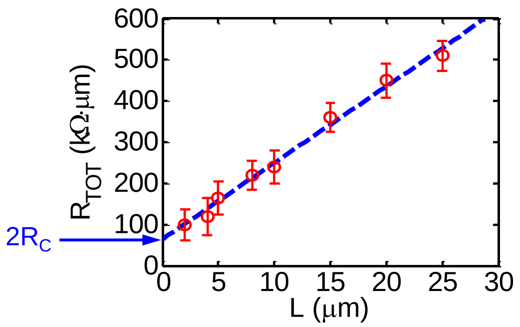

Figure S7 | Transfer length method (TLM). Electrical resistance (normalized by width) of our GST test structures with varying channel length to obtain contact and sheet resistivity. The y-axis intercept represents the resistance of the contacts ($2R_C$), and the sheet resistance can be obtained from the slope.

8. PCM device breakdown

The lateral PCM devices undergo breakdown at high electrical bias, rather than reset to amorphous state. These devices degrade when the GST channel is self-heated, ostensibly because the PMMA capping layer evaporates, leaving the GST exposed to air at high temperature, at which it oxidizes. Optical images of the devices post-breakdown reveal damage to the GST film near the ground

terminal, as shown in Fig. S8(a). The location of the breakdown spot agrees well with the measured peak temperature [Fig. S8(b)].

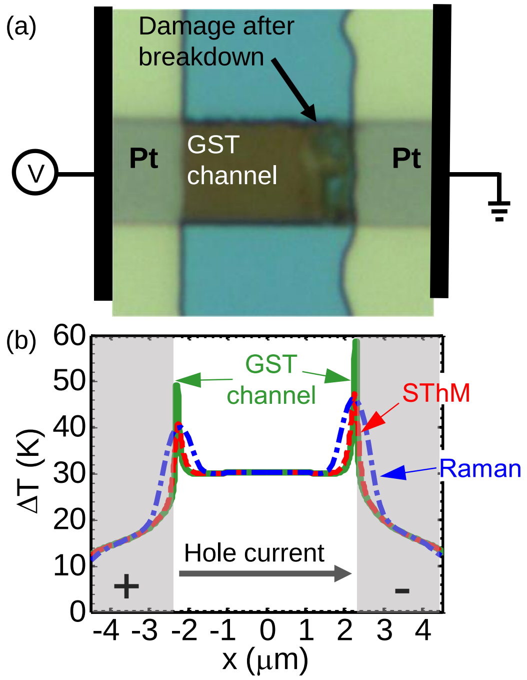

Figure S8 | Post-breakdown damage. (a) Optical image of GST channel after breakdown showing damage near the grounded electrode. (b) Simulated temperature profile (calibrated by measurements) of the GST channel (see Fig. 5 in the main text for details) showing the peak temperature located near the grounded terminal.

9. Parameters used in COMSOL simulation

Table I – parameters used in finite element modeling simulations (near room temperature)

Parameter	Description	Value	Reference\comments
ρ_{GST}	GST Electrical specific resistivity	35 mΩ·cm	TLM measurement (fcc)
$\rho_{C,GST}$	Electrical contact resistivity between GST-Pt	$4\cdot10^{-4}$ Ω·cm^2	TLM measurement (fcc)
ρ_{Pt}	Pt Electrical specific resistivity	$1.8\cdot10^{-7}$ Ω·cm	4-probe measurement of similar films
k_{GST}	GST thermal conductivity	1 Wm^{-1}K^{-1}	We set k_{GST} = 1 Wm^{-1}K^{-1}, (Ref. 17) but the simulated temperature rise in the lateral device is relatively insensitive (less than 10% change) to thermal conductivity changes in the range k_{GST} ~ 0.5 to 1.5 Wm^{-1}K^{-1}
k_{Pt}	Pt thermal conductivity	50 Wm^{-1}K^{-1}	Estimated from measured ρ_{Pt} via

			Wiedemann Franz law
k_{PMMA}	PMMA thermal conductivity	0.19 $Wm^{-1}K^{-1}$	COMSOL materials library<br>Steady state temperature profile is insensitive to this parameter
k_{SiO2}	SiO_2 thermal conductivity	1.4 $Wm^{-1}K^{-1}$	Ref. 18
k_{Si}	Si (p^{++}) thermal conductivity	95 $Wm^{-1}K^{-1}$	Ref. 5
TBR GST-SiO_2	Thermal boundary resistance (TBR) of GST-SiO_2 interface	28 m^2K/GW	Determined by fitting the temperature at the center of the channel to experimental data. Error ±8 m^2K/GW
TBR GST-Pt	TBR of GST-Pt interface	25 m^2K/GW	Determined by fitting the temperature profile at the contact area to experimental data. Error ±9 m^2K/GW
TBR Si-SiO_2	TBR of Si-SiO_2 interface	3 m^2K/GW	Ref. 18
TBR GST-PMMA	TBR of GST-PMMA interface	25 m^2K/GW	Set to similar value as GST-Pt, but steady state temperature profile is insensitive to this parameter

Supplementary References

1 Schadler, L. & Galiotis, C. Fundamentals and applications of micro Raman spectroscopy to strain measurements in fibre reinforced composites. *International Materials Reviews* **40**, 116-134 (1995).
2 Basko, D., Piscanec, S. & Ferrari, A. Electron-electron interactions and doping dependence of the two-phonon Raman intensity in graphene. *Physical Review B* **80**, 165413 (2009).
3 Dresselhaus, M., Jorio, A., Cançado, L., Dresselhaus, G. & Saito, R. in *Graphene Nanoelectronics* 15-55 (Springer, 2011).
4 Freitag, M., Steiner, M., Martin, Y., Perebeinos, V., Chen, Z., Tsang, J. C. & Avouris, P. Energy dissipation in graphene field-effect transistors. *Nano Letters* **9**, 1883-1888 (2009).
5 Yalon, E., McClellan, C. J., Smithe, K. K., Munoz-Rojo, M., Xu, R., Suryavanshi, S. V., Gabourie, A. J., Neumann, C. M., Xiong, F., Farimani, A. B. & Pop, E. Energy Dissipation in Monolayer MoS_2 Electronics. *Nano Letters* **17**, 3429–3433 (2017).
6 Kip, B. J. & Meier, R. J. Determination of the local temperature at a sample during Raman experiments using Stokes and anti-Stokes Raman bands. *Applied Spectroscopy* **44**, 707-711 (1990).
7 Tsai, C.-L., Liao, A., Pop, E. & Shim, M. Electrical power dissipation in semiconducting carbon nanotubes on single crystal quartz and amorphous SiO2. *Applied Physics Letters* **99**, 053120 (2011).
8 Pine, A. & Dresselhaus, G. Raman spectra and lattice dynamics of tellurium. *Physical Review B* **4**, 356 (1971).
9 Majumdar, A. Scanning thermal microscopy. *Annual Review of Materials Science* **29**, 505-585 (1999).

10 Borca-Tasciuc, T. Scanning probe methods for thermal and thermoelectric property measurements. *Annu. Rev. Heat Transfer* **16**, 211-258 (2013).

11 Wilson, A. A., Rojo, M. M., Abad, B., Perez, J. A., Maiz, J., Schomacker, J., Martín-Gonzalez, M., Borca-Tasciuc, D.-A. & Borca-Tasciuc, T. Thermal conductivity measurements of high and low thermal conductivity films using a scanning hot probe method in the 3 ω mode and novel calibration strategies. *Nanoscale* **7**, 15404-15412 (2015).

12 Volz, S., Feng, X., Fuentes, C., Guérin, P. & Jaouen, M. Thermal conductivity measurements of thin amorphous silicon films by scanning thermal microscopy. *International Journal of Thermophysics* **23**, 1645-1657 (2002).

13 Muñoz Rojo, M., Grauby, S., Rampnoux, J.-M., Caballero-Calero, O., Martin-Gonzalez, M. & Dilhaire, S. Fabrication of Bi2Te3 nanowire arrays and thermal conductivity measurement by 3ω-scanning thermal microscopy. *Journal of Applied Physics* **113**, 054308 (2013).

14 Rojo, M. M., Martín, J., Grauby, S., Borca-Tasciuc, T., Dilhaire, S. & Martin-Gonzalez, M. Decrease in thermal conductivity in polymeric P3HT nanowires by size-reduction induced by crystal orientation: new approaches towards thermal transport engineering of organic materials. *Nanoscale* **6**, 7858-7865 (2014).

15 Menges, F., Mensch, P., Schmid, H., Riel, H., Stemmer, A. & Gotsmann, B. Temperature mapping of operating nanoscale devices by scanning probe thermometry. *Nature Communications* **7**, 10874 (2016).

16 Roy, D., in't Zandt, M., Wolters, R., Timmering, C. & Klootwijk, J. in *2009 Non-Volatile Memory Technology Symposium (NVMTS).* 12-15 (IEEE, DOI:10.1109/NVMT.2009.5429780).

17 Reifenberg, J. P., Panzer, M. A., Kim, S., Gibby, A. M., Zhang, Y., Wong, S., Wong, H.-S. P., Pop, E. & Goodson, K. E. Thickness and stoichiometry dependence of the thermal conductivity of GeSbTe films. *Applied Physics Letters* **91**, 111904 (2007).

18 Chien, H.-C., Yao, D.-J., Huang, M.-J. & Chang, T.-Y. Thermal conductivity measurement and interface thermal resistance estimation using SiO_2 thin film. *Review of Scientific Instruments* **79**, 054902 (2008).
